# Lessons learned from organizing and evaluating international virtual training for healthcare professionals

**DOI:** 10.5116/ijme.6238.459f

**Published:** 2022-03-31

**Authors:** Tamara Galoyan, Lynn Kysh, Armine Lulejian, James Dickhoner, Abu Sikder, Mindy Lee, Eyal Ben-Isaac, Juan Espinoza

**Affiliations:** 1 School of Education, Drexel University, USA; 2 Institute for Nursing and Interprofessional Research (INIR), Children's Hospital Los Angeles, USA; 3 Keck School of Medicine, University of Southern California, USA; 4 Innovation Studio, Children's Hospital Los Angeles, USA; 5 Department of Pediatrics, Children's Hospital Los Angeles, USA

**Keywords:** Lessons learned, evaluation, international virtual training, healthcare professionals

## To the Editor

Education is a critical part of capacity building in global health partnerships.^1^ Medical training conferences designed for providers in low-to-middle-income countries (LMICs) address equity in global health by reducing financial, political, and language barriers.^2^ However, the COVID-19 pandemic has disrupted gatherings, added further travel restrictions, and exhausted already strained financial resources for individuals from LMICs to attend conferences. To continue providing quality education, training, and professional development in the midst of the global crisis, organizations had to rapidly transition to virtual platforms.^3^^,^^4^ The pandemic has accelerated the process of adoption of innovative technological solutions, including learning management systems (LMS) (e.g., Moodle, Canvas) and virtual meeting platforms (e.g., Zoom, Google Hangouts, GoToMeeting) that transcend geographic and temporal boundaries, enhancing the potential of reaching a wider audience. This has also had the benefit of addressing some of the preexisting barriers for conference attendees from LMICs.

This transition was not unprecedented; as early as 2009, The US Commitment to Global Health: Recommendations for the Public and Private Sectors report by the US Institute of Medicine^5^ created a framework to guide investments and implementation in global health, including sharing knowledge, investing in people and institutions, and engaging in respectful partnerships. Virtual international meetings have been held successfully prior to the pandemic in response to environmental concerns and financial limitations.^6^^-^^10^

Prior studies documented the benefits of utilizing online formats for medical education, including training healthcare professionals via virtual conferences.^11^ Among those benefits include saving time, resources, and money.^11^^,^^12^ In addition, virtual conferences help healthcare associations and universities increase their global outreach by forming international partnerships and collaborations, as well as disseminating their research at a global scale.^11^ Despite their benefits of online and virtual formats, researchers indicate that evaluating the effectiveness of online platforms for medical education still remains a challenge.^12^^,^^13^

To address the challenge, our multidisciplinary team at Children's Hospital Los Angeles (CHLA) partnered with the Armenian Eye Care Project (AECP) to co-host a series of three international virtual training conferences on ophthalmology and pediatrics for healthcare professionals in the Republic of Armenia and its surrounding regions between September 2020 and February 2021. The conference planning began in December 2019. Due to the COVID-19 pandemic, we transitioned to a fully virtual format in June 2020. Our evaluation strategy included a collection of quantitative and qualitative data from the conference participants through a post-conference survey and semi-structured interviews on their experiences and perceptions. Below, we are sharing the lessons learned from the process of organizing and evaluating virtual training conferences.

As to positive experiences, we learned that, overall, participant experiences with the conference were positive. They appreciated having practical, well-selected, and highly educational topics, well-delivered lectures, and opportunities to network and connect with foreign colleagues. Most of them also indicated that they would recommend such conferences to their colleagues in the field. Our findings are in alignment with previous studies documenting the benefits of virtual formats for medical education and training.^11^^,^^12^

Regarding the benefits of the virtual format, conducting conferences in a virtual format has several benefits both from the organizers and the participants' standpoints. In alignment with the recent studies showcasing the benefits of conducting medical educational training in virtual platforms,^11^ our findings suggest that utilizing the virtual platforms can help to break the time and distance barriers to reach a wider audience and make the content accessible and engaging.

On application of knowledge and skills, we learned that our participants benefited from the conference in terms of the opportunities to transfer the learned knowledge and skills to their practice. Most of our participants saw the potential application of knowledge and skills to practice as one of the most valuable features of the conferences.

Concerning preference for native language, we learned that the language barrier could be an issue when organizing an international conference in countries where English is considered a foreign language. We found that our participants preferred to attend the conference in their native language, Armenian.

With regard to participant recommendations, feedback collected from participants in the form of qualitative data from both the open-ended survey items and the follow-up interviews helped the conference organizers identify several helpful recommendations for future conferences. For instance, some of the suggestions related to including additional lecture topics (e.g., immunology, pediatric pulmonology, rheumatology, infectious diseases, asthma, etc.) and surveying the Armenian doctors to identify lecture topics, some others included holding the conferences more frequently, having more interactive sessions, including Q&A and debates, and inviting speakers from other countries.

Regarding implications for future research and practice, our experiences align with previous studies showcasing the positive outcomes of organizing virtual professional development training for healthcare professionals.^6^^-^^8^ As a next step, our team will use the key lessons learned from this study to plan, implement, and evaluate future medical education training conferences with a hybrid approach (online supplemented with face-to-face components). This will allow the team to build upon the strengths of both formats to enhance the quality of learner experiences. In particular, we are planning to experiment with more interactive elements, including more networking time, additional Q&A slots, and more webinars to increase the frequency of educational events. Furthermore, to improve the quality of our evaluation and to increase participation, we are planning to disseminate the survey and conduct interviews closer to the end of the conference to increase response rates and survey people while the experience is still fresh. Further research is needed to explore and compare experiences and perceptions of medical training conferences across participant demographics, learning content, and instructional modalities, including online, hybrid, and face-to-face.

In summary, the lessons learned from our experience with organizing and evaluating virtual training conferences for healthcare professionals in LMICs can be adapted by medical institutions to organize virtual training in other areas of medical education. Adoption and application of effective educational practices for virtual formats are especially important considering the worldwide disruptions caused by the COVID-19 pandemic. As the future of large in-person conferences remains unclear, universities and healthcare associations must provide access to integrated and high-quality virtual meetings to continue facilitating knowledge and skills development for healthcare professionals worldwide.

### Conflict of Interest

The authors declare that they have no potential conflict of interest.
